# Crystal structure of ethyl 2-amino-4-(4-meth­oxy­phen­yl)-4*H*-1-benzothieno[3,2-*b*]pyran-3-carboxyl­ate

**DOI:** 10.1107/S2056989015008154

**Published:** 2015-04-30

**Authors:** Mohamed Bakhouch, Abdelali Kerbal, Mohamed El Yazidi, Mohamed Saadi, Lahcen El Ammari

**Affiliations:** a Département de Chimie, Faculté des Sciences, Dhar Mehraz, BP 1796 Atlas, 30000 Fes, Morocco; b Laboratoire de Chimie du Solide Appliquée, Faculté des Sciences, Université Mohammed V, Avenue Ibn Battouta, BP 1014, Rabat, Morocco

**Keywords:** crystal structure, thio­aurones, hydrogen bonding

## Abstract

The mol­ecule of the title compound, C_21_H_19_NO_4_S, features a fused ring system whereby a five-membered ring is flanked by two six-membered rings. This is linked to an ethyl 3-carboxyl­ate group and to a meth­oxy­benzene group. The fused-ring system is quasi-planar, with the greatest deviation from the mean plane being 0.131 (1) Å for the methine C atom. The plane through the meth­oxy­benzene ring is nearly perpendicular to that through the fused-ring system, as indicated by the dihedral angle of 85.72 (6)°. An intra­molecular N—H⋯O hydrogen bond is noted. In the crystal, mol­ecules are linked by N—H⋯O hydrogen bonds, forming layers that stack along the *a* axis.

## Related literature   

For biological properties of substituted 2-amino-4-aryl-4*H*-pyran derivatives, see: Panda *et al.* (1997 ▸); Mungra *et al.* (2011 ▸). For the reactivity of (*Z*)-2-aryl­idenebenzo[*b*]thio­phen-3(2*H*)-ones (thio­aurones), see: Boughaleb *et al.* (2010 ▸; 2011 ▸); Bakhouch *et al.* (2014 ▸, 2015 ▸). For the preparation of the title compound, using condensation reactions, see: Daisley *et al.* (1982 ▸).
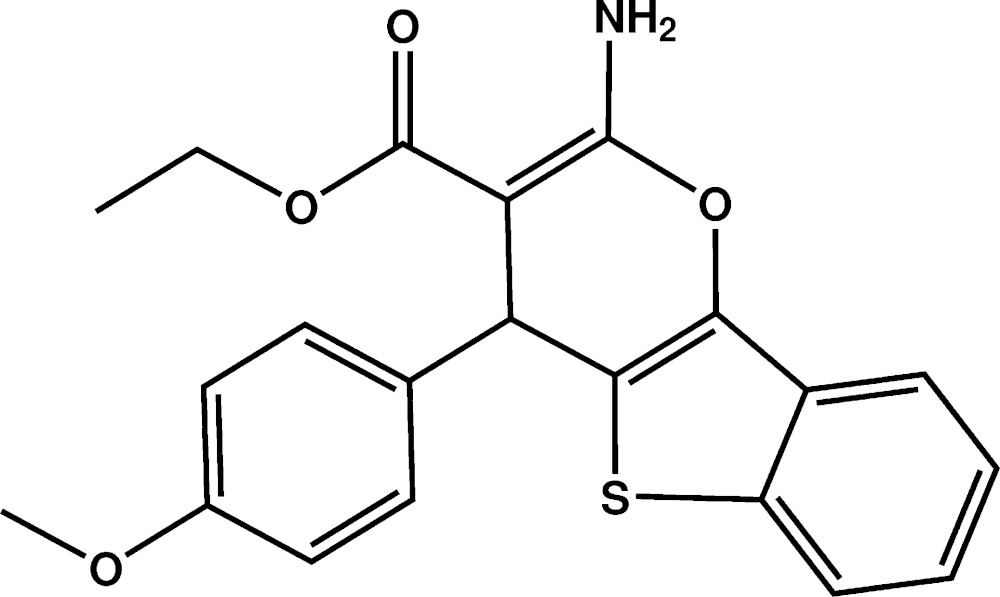


## Experimental   

### Crystal data  

C_21_H_19_NO_4_S
*M* *_r_* = 381.43
Monoclinic, 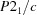

*a* = 11.8355 (7) Å
*b* = 18.6222 (11) Å
*c* = 9.0110 (5) Å
β = 106.502 (2)°
*V* = 1904.25 (19) Å^3^
*Z* = 4
Mo *K*α radiation
μ = 0.20 mm^−1^
*T* = 296 K
0.42 × 0.31 × 0.26 mm

### Data collection  

Bruker X8 APEX diffractometer
Absorption correction: multi-scan (*SADABS*; Sheldrick, 1995 ▸) *T*_min_ = 0.673, *T*_max_ = 0.746
38870 measured reflections
5347 independent reflections
4118 reflections with *I* > 2σ(*I*)
*R*_int_ = 0.031

### Refinement  

*R*[*F* ^2^ > 2σ(*F* ^2^)] = 0.044
*wR*(*F* ^2^) = 0.130
*S* = 1.06
5347 reflections
244 parameters
H-atom parameters constrained
Δρ_max_ = 0.25 e Å^−3^
Δρ_min_ = −0.27 e Å^−3^

### 

Data collection: *APEX2* (Bruker, 2009 ▸); cell refinement: *SAINT* (Bruker, 2009 ▸); data reduction: *SAINT*; program(s) used to solve structure: *SHELXS97* (Sheldrick, 2008 ▸); program(s) used to refine structure: *SHELXL97* (Sheldrick, 2008 ▸); molecular graphics: *ORTEP-3 for Windows* (Farrugia, 2012 ▸); software used to prepare material for publication: *PLATON* (Spek, 2009 ▸) and *publCIF* (Westrip, 2010 ▸).

## Supplementary Material

Crystal structure: contains datablock(s) I. DOI: 10.1107/S2056989015008154/tk5366sup1.cif

Structure factors: contains datablock(s) I. DOI: 10.1107/S2056989015008154/tk5366Isup2.hkl

Supporting information file. DOI: 10.1107/S2056989015008154/tk5366Isup3.cml

**Fig. 1.**
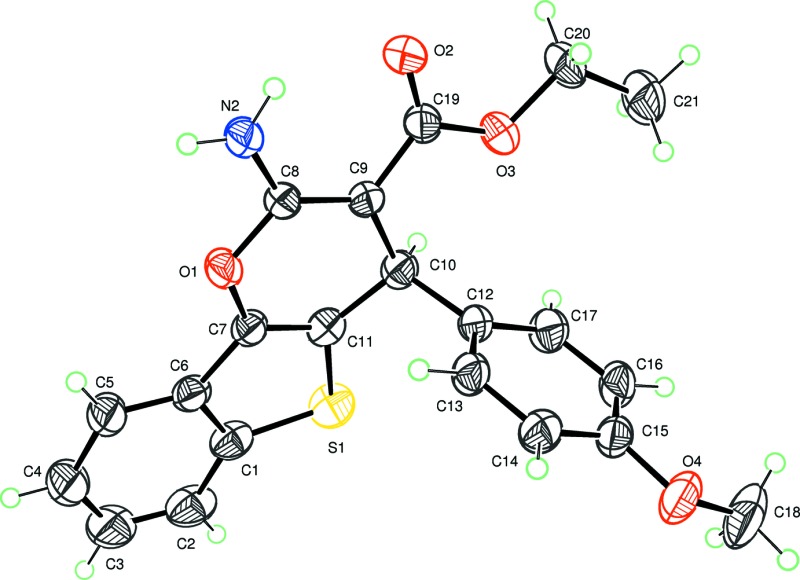
Plot of the molecule of the title compound with the atom-labelling scheme. Displacement ellipsoids are drawn at the 50% probability level. H atoms are represented as small circles.

. DOI: 10.1107/S2056989015008154/tk5366fig1.tif
Plot of the mol­ecule of the title compound with the atom-labelling scheme. Displacement ellipsoids are drawn at the 50% probability level. H atoms are represented as small circles.

CCDC reference: 1061576

Additional supporting information:  crystallographic information; 3D view; checkCIF report

## Figures and Tables

**Table 1:** Hydrogen-bond geometry (, )

*D*H*A*	*D*H	H*A*	*D* *A*	*D*H*A*
N2H2*B*O2	0.86	2.08	2.6903(16)	128
N2H2*A*O2^i^	0.86	2.17	2.9964(16)	161
N2H2*B*O4^ii^	0.86	2.38	3.0908(17)	140

Symmetry codes: (i) 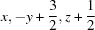; (ii) 
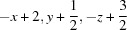
.

## supplementary crystallographic information

### S1. Comment

Substituted 2-amino-4-aryl-4*H*-pyran derivatives are an important class of heterocyclic compounds, which frequently exhibit a wide range of biological properties *viz*. antiproliferative, antitubercular activities (Panda *et al.*, 1997; Mungra *et al.*, 2011). Thus, in view of the large spectrum of applications of these compounds and in continuation of ongoing research focused on the reactivity of the (*Z*)-2-arylidenebenzo[*b*]thiophen- 3(2*H*)-ones (thioaurones) (Boughaleb *et al.*, 2010; 2011; Bakhouch *et al.*, 2014; 2015), the title compound was investigated This was prepared by the the action of ethyl cyanoacetate on (*Z*)-2-(4- methoxybenzylidene)benzo[*b*]thiophen-3(2*H*)-one (Daisley *et al.*, 1982). The subsequent tautomeric transformation gives rise to ethyl 2-amino-4-(4-methoxyphenyl)-4*H*-1-benzothieno[3,2-*b*]pyran-3- carboxylate.

The molecule of the title compound is formed by three fused rings linked to an ethyl-3-carboxylate group and to a methoxybenzene group as shown in Fig. 1. The three fused rings (S1C1 to C11 O1) are nearly coplanar, with the maximum deviation from the mean plane being -0.131 (1) Å at C10, and makes a dihedral angle of 85.72 (6)° with the plans through this attached methoxyphenyl group.

In the crystal, the molecules are linked by N—H···O hydrogen bonds as shown in Fig. 2 and Table 1.

### S2. Experimental

In a 100 ml flask equipped with a condenser was dissolved 4 mmol of (*Z*)-2-(4-methoxybenzylidene)-1-benzo[*b*]thiophen-3(2*H*)-one and 5 mmol of ethyl cyanoacetate in 30 ml of ethanol. Then, 1 ml of piperidine was added, and the reaction mixture was refluxed for 6 h. Thin layer chromatography revealed the formation of a single product. The organic phase was evaporated under reduced pressure. The resulting residue was recrystallized from ethanol by slow evaporation (Yield: 28%; m.pt: 395 K).

### S3. Refinement

H atoms were located in a difference map and treated as riding with C—H = 0.93–0.98 Å and N—H = 0.86 Å, and with *U*_iso_(H) = 1.2–1.5 *U*_eq_(C) and *U*_iso_(H) = 1.2 *U*_eq_(N). Two reflections, *i.e*. (1 0 0) and (1 1 0), were omitted from the final refinement owing to poor agreement.

### Crystal data

**Table:**

C_21_H_19_NO_4_S	*F*(000) = 800
*M**_r_* = 381.43	*D*_x_ = 1.330 Mg m^−^^3^
Monoclinic, *P*2_1_/*c*	Mo *K*α radiation, λ = 0.71073 Å
Hall symbol: -P 2ybc	Cell parameters from 5347 reflections
*a* = 11.8355 (7) Å	θ = 2.2–29.6°
*b* = 18.6222 (11) Å	µ = 0.20 mm^−^^1^
*c* = 9.0110 (5) Å	*T* = 296 K
β = 106.502 (2)°	Block, colourless
*V* = 1904.25 (19) Å^3^	0.42 × 0.31 × 0.26 mm
*Z* = 4	

### Data collection

**Table:**

Bruker X8 APEX diffractometer	5347 independent reflections
Radiation source: fine-focus sealed tube	4118 reflections with *I* > 2σ(*I*)
Graphite monochromator	*R*_int_ = 0.031
φ and ω scans	θ_max_ = 29.6°, θ_min_ = 2.2°
Absorption correction: multi-scan (*SADABS*; Sheldrick, 1995)	*h* = −16→16
*T*_min_ = 0.673, *T*_max_ = 0.746	*k* = −25→25
38870 measured reflections	*l* = −8→12

### Refinement

**Table:**

Refinement on *F*^2^	Primary atom site location: structure-invariant direct methods
Least-squares matrix: full	Secondary atom site location: difference Fourier map
*R*[*F*^2^ > 2σ(*F*^2^)] = 0.044	Hydrogen site location: inferred from neighbouring sites
*wR*(*F*^2^) = 0.130	H-atom parameters constrained
*S* = 1.06	*w* = 1/[σ^2^(*F*_o_^2^) + (0.0559*P*)^2^ + 0.5166*P*] where *P* = (*F*_o_^2^ + 2*F*_c_^2^)/3
5347 reflections	(Δ/σ)_max_ = 0.001
244 parameters	Δρ_max_ = 0.25 e Å^−^^3^
0 restraints	Δρ_min_ = −0.27 e Å^−^^3^

### Special details

**Table:**

Geometry. All e.s.d.'s (except the e.s.d. in the dihedral angle between two l.s. planes) are estimated using the full covariance matrix. The cell e.s.d.'s are taken into account individually in the estimation of e.s.d.'s in distances, angles and torsion angles; correlations between e.s.d.'s in cell parameters are only used when they are defined by crystal symmetry. An approximate (isotropic) treatment of cell e.s.d.'s is used for estimating e.s.d.'s involving l.s. planes.
Refinement. Refinement of *F*^2^ against all reflections. The weighted *R*-factor *wR* and goodness of fit *S* are based on *F*^2^, conventional *R*-factors *R* are based on *F*, with *F* set to zero for negative *F*^2^. The threshold expression of *F*^2^ > σ(*F*^2^) is used only for calculating *R*-factors(gt) *etc*. and is not relevant to the choice of reflections for refinement. *R*-factors based on *F*^2^ are statistically about twice as large as those based on *F*, and *R*- factors based on all data will be even larger.

### Fractional atomic coordinates and isotropic or equivalent isotropic displacement parameters (Å^2^)

**Table:**

	*x*	*y*	*z*	*U*_iso_*/*U*_eq_	
C1	0.54915 (13)	0.61283 (8)	0.9681 (2)	0.0525 (4)	
C2	0.46963 (16)	0.61032 (10)	1.0560 (3)	0.0699 (5)	
H2	0.4006	0.5837	1.0231	0.084*	
C3	0.4953 (2)	0.64792 (11)	1.1919 (3)	0.0816 (6)	
H3	0.4434	0.6463	1.2522	0.098*	
C4	0.5973 (2)	0.68840 (11)	1.2411 (3)	0.0830 (7)	
H4	0.6126	0.7137	1.3337	0.100*	
C5	0.67644 (18)	0.69176 (9)	1.1550 (2)	0.0666 (5)	
H5	0.7445	0.7193	1.1885	0.080*	
C6	0.65300 (13)	0.65344 (7)	1.01733 (18)	0.0460 (3)	
C7	0.72073 (12)	0.64514 (7)	0.90952 (16)	0.0412 (3)	
C8	0.88161 (12)	0.67389 (7)	0.81926 (15)	0.0394 (3)	
C9	0.84330 (12)	0.62861 (7)	0.69542 (15)	0.0405 (3)	
C10	0.73518 (13)	0.58116 (7)	0.67067 (16)	0.0434 (3)	
H10	0.6822	0.5911	0.5675	0.052*	
C11	0.67418 (12)	0.60232 (7)	0.78831 (17)	0.0436 (3)	
C12	0.76078 (13)	0.50065 (7)	0.68312 (15)	0.0416 (3)	
C13	0.83473 (14)	0.47133 (8)	0.81661 (16)	0.0484 (3)	
H13	0.8741	0.5016	0.8968	0.058*	
C14	0.85118 (15)	0.39799 (8)	0.83308 (19)	0.0537 (4)	
H14	0.9009	0.3792	0.9239	0.064*	
C15	0.79345 (14)	0.35251 (8)	0.7142 (2)	0.0517 (4)	
C16	0.72156 (16)	0.38052 (9)	0.5795 (2)	0.0587 (4)	
H16	0.6839	0.3503	0.4983	0.070*	
C17	0.70549 (15)	0.45444 (9)	0.56549 (18)	0.0547 (4)	
H17	0.6561	0.4732	0.4743	0.066*	
C18	0.7396 (2)	0.23146 (10)	0.6393 (4)	0.0980 (8)	
H18A	0.7623	0.1832	0.6719	0.147*	
H18B	0.7473	0.2389	0.5372	0.147*	
H18C	0.6592	0.2392	0.6384	0.147*	
C19	0.90696 (14)	0.62770 (7)	0.58012 (16)	0.0462 (3)	
C20	0.9195 (2)	0.56999 (10)	0.3491 (2)	0.0703 (5)	
H20A	1.0017	0.5579	0.3960	0.084*	
H20B	0.9156	0.6133	0.2881	0.084*	
C21	0.8570 (3)	0.50952 (12)	0.2497 (2)	0.0897 (7)	
H21A	0.8935	0.5004	0.1691	0.135*	
H21B	0.7758	0.5223	0.2045	0.135*	
H21C	0.8614	0.4672	0.3118	0.135*	
N2	0.97404 (11)	0.71791 (7)	0.84860 (15)	0.0496 (3)	
H2A	0.9907	0.7445	0.9300	0.060*	
H2B	1.0171	0.7197	0.7862	0.060*	
O1	0.82701 (9)	0.68022 (6)	0.93374 (12)	0.0483 (2)	
O2	0.99346 (11)	0.66385 (6)	0.58006 (13)	0.0556 (3)	
O3	0.85945 (12)	0.58024 (6)	0.46707 (12)	0.0600 (3)	
O4	0.81361 (12)	0.28053 (6)	0.74361 (18)	0.0727 (4)	
S1	0.53964 (4)	0.56789 (3)	0.79591 (6)	0.06281 (15)	

### Atomic displacement parameters (Å^2^)

**Table:**

	*U*^11^	*U*^22^	*U*^33^	*U*^12^	*U*^13^	*U*^23^
C1	0.0427 (7)	0.0455 (7)	0.0715 (10)	0.0012 (6)	0.0199 (7)	0.0062 (7)
C2	0.0508 (9)	0.0631 (10)	0.1059 (15)	−0.0028 (8)	0.0388 (10)	0.0102 (10)
C3	0.0858 (14)	0.0717 (12)	0.1146 (18)	−0.0017 (10)	0.0725 (14)	0.0004 (12)
C4	0.1067 (16)	0.0694 (12)	0.1008 (16)	−0.0151 (11)	0.0746 (14)	−0.0204 (11)
C5	0.0791 (12)	0.0565 (9)	0.0813 (12)	−0.0179 (8)	0.0506 (10)	−0.0193 (9)
C6	0.0471 (7)	0.0364 (6)	0.0608 (9)	−0.0011 (5)	0.0253 (6)	0.0012 (6)
C7	0.0417 (6)	0.0352 (6)	0.0491 (7)	−0.0029 (5)	0.0169 (6)	−0.0019 (5)
C8	0.0445 (6)	0.0358 (6)	0.0414 (7)	0.0006 (5)	0.0181 (5)	0.0004 (5)
C9	0.0516 (7)	0.0336 (6)	0.0379 (7)	0.0003 (5)	0.0150 (6)	0.0020 (5)
C10	0.0501 (7)	0.0408 (6)	0.0350 (6)	−0.0021 (5)	0.0053 (5)	−0.0008 (5)
C11	0.0412 (6)	0.0392 (6)	0.0483 (8)	−0.0011 (5)	0.0093 (6)	0.0010 (5)
C12	0.0507 (7)	0.0392 (6)	0.0347 (6)	−0.0062 (5)	0.0120 (5)	−0.0052 (5)
C13	0.0635 (9)	0.0413 (7)	0.0368 (7)	−0.0032 (6)	0.0086 (6)	−0.0061 (5)
C14	0.0648 (9)	0.0446 (8)	0.0515 (9)	0.0040 (7)	0.0162 (7)	0.0033 (6)
C15	0.0564 (8)	0.0375 (7)	0.0727 (10)	−0.0054 (6)	0.0370 (8)	−0.0095 (6)
C16	0.0681 (10)	0.0511 (8)	0.0597 (10)	−0.0175 (7)	0.0230 (8)	−0.0239 (7)
C17	0.0639 (9)	0.0534 (8)	0.0417 (8)	−0.0105 (7)	0.0068 (7)	−0.0106 (6)
C18	0.0876 (14)	0.0439 (9)	0.175 (3)	−0.0152 (9)	0.0570 (16)	−0.0330 (13)
C19	0.0666 (9)	0.0370 (6)	0.0378 (7)	0.0055 (6)	0.0192 (6)	0.0035 (5)
C20	0.1086 (16)	0.0698 (11)	0.0429 (9)	0.0043 (10)	0.0384 (10)	−0.0031 (7)
C21	0.146 (2)	0.0812 (14)	0.0477 (10)	−0.0027 (14)	0.0360 (12)	−0.0168 (9)
N2	0.0545 (7)	0.0493 (6)	0.0527 (7)	−0.0134 (5)	0.0276 (6)	−0.0125 (5)
O1	0.0499 (5)	0.0532 (6)	0.0486 (6)	−0.0163 (4)	0.0248 (4)	−0.0154 (4)
O2	0.0742 (7)	0.0480 (6)	0.0545 (6)	−0.0039 (5)	0.0345 (6)	0.0020 (5)
O3	0.0858 (8)	0.0595 (6)	0.0406 (6)	−0.0071 (6)	0.0277 (6)	−0.0085 (5)
O4	0.0800 (8)	0.0368 (5)	0.1128 (11)	−0.0022 (5)	0.0458 (8)	−0.0092 (6)
S1	0.0452 (2)	0.0688 (3)	0.0704 (3)	−0.01563 (18)	0.00995 (19)	−0.0072 (2)

### Geometric parameters (Å, º)

**Table:**

C1—C2	1.393 (2)	C13—C14	1.382 (2)
C1—C6	1.404 (2)	C13—H13	0.9300
C1—S1	1.7381 (18)	C14—C15	1.384 (2)
C2—C3	1.367 (3)	C14—H14	0.9300
C2—H2	0.9300	C15—C16	1.372 (3)
C3—C4	1.385 (3)	C15—O4	1.3742 (18)
C3—H3	0.9300	C16—C17	1.390 (2)
C4—C5	1.377 (2)	C16—H16	0.9300
C4—H4	0.9300	C17—H17	0.9300
C5—C6	1.389 (2)	C18—O4	1.420 (3)
C5—H5	0.9300	C18—H18A	0.9600
C6—C7	1.4327 (19)	C18—H18B	0.9600
C7—C11	1.3381 (19)	C18—H18C	0.9600
C7—O1	1.3786 (16)	C19—O2	1.2255 (19)
C8—N2	1.3319 (17)	C19—O3	1.3451 (18)
C8—O1	1.3691 (15)	C20—O3	1.450 (2)
C8—C9	1.3691 (18)	C20—C21	1.497 (3)
C9—C19	1.4464 (19)	C20—H20A	0.9700
C9—C10	1.5185 (19)	C20—H20B	0.9700
C10—C11	1.495 (2)	C21—H21A	0.9600
C10—C12	1.5273 (19)	C21—H21B	0.9600
C10—H10	0.9800	C21—H21C	0.9600
C11—S1	1.7360 (14)	N2—H2A	0.8600
C12—C17	1.3778 (19)	N2—H2B	0.8600
C12—C13	1.383 (2)		
			
C2—C1—C6	120.72 (17)	C12—C13—H13	119.4
C2—C1—S1	127.51 (14)	C13—C14—C15	119.88 (15)
C6—C1—S1	111.75 (12)	C13—C14—H14	120.1
C3—C2—C1	118.55 (17)	C15—C14—H14	120.1
C3—C2—H2	120.7	C16—C15—O4	124.87 (15)
C1—C2—H2	120.7	C16—C15—C14	119.85 (14)
C2—C3—C4	121.17 (17)	O4—C15—C14	115.28 (16)
C2—C3—H3	119.4	C15—C16—C17	119.43 (14)
C4—C3—H3	119.4	C15—C16—H16	120.3
C5—C4—C3	120.97 (19)	C17—C16—H16	120.3
C5—C4—H4	119.5	C12—C17—C16	121.70 (15)
C3—C4—H4	119.5	C12—C17—H17	119.1
C4—C5—C6	118.99 (18)	C16—C17—H17	119.1
C4—C5—H5	120.5	O4—C18—H18A	109.5
C6—C5—H5	120.5	O4—C18—H18B	109.5
C5—C6—C1	119.60 (14)	H18A—C18—H18B	109.5
C5—C6—C7	130.75 (14)	O4—C18—H18C	109.5
C1—C6—C7	109.63 (13)	H18A—C18—H18C	109.5
C11—C7—O1	123.86 (12)	H18B—C18—H18C	109.5
C11—C7—C6	115.96 (13)	O2—C19—O3	122.02 (13)
O1—C7—C6	120.17 (12)	O2—C19—C9	126.84 (13)
N2—C8—O1	109.53 (11)	O3—C19—C9	111.15 (13)
N2—C8—C9	127.16 (12)	O3—C20—C21	105.98 (17)
O1—C8—C9	123.31 (12)	O3—C20—H20A	110.5
C8—C9—C19	118.24 (12)	C21—C20—H20A	110.5
C8—C9—C10	123.25 (12)	O3—C20—H20B	110.5
C19—C9—C10	118.48 (12)	C21—C20—H20B	110.5
C11—C10—C9	107.39 (11)	H20A—C20—H20B	108.7
C11—C10—C12	109.41 (11)	C20—C21—H21A	109.5
C9—C10—C12	114.78 (12)	C20—C21—H21B	109.5
C11—C10—H10	108.4	H21A—C21—H21B	109.5
C9—C10—H10	108.4	C20—C21—H21C	109.5
C12—C10—H10	108.4	H21A—C21—H21C	109.5
C7—C11—C10	124.48 (13)	H21B—C21—H21C	109.5
C7—C11—S1	110.95 (11)	C8—N2—H2A	120.0
C10—C11—S1	124.31 (10)	C8—N2—H2B	120.0
C17—C12—C13	117.87 (13)	H2A—N2—H2B	120.0
C17—C12—C10	121.04 (13)	C8—O1—C7	116.40 (10)
C13—C12—C10	120.98 (12)	C19—O3—C20	117.08 (14)
C14—C13—C12	121.25 (13)	C15—O4—C18	117.34 (17)
C14—C13—H13	119.4	C11—S1—C1	91.71 (7)

### Hydrogen-bond geometry (Å, º)

**Table:**

*D*—H···*A*	*D*—H	H···*A*	*D*···*A*	*D*—H···*A*
N2—H2*B*···O2	0.86	2.08	2.6903 (16)	128
N2—H2*A*···O2^i^	0.86	2.17	2.9964 (16)	161
N2—H2*B*···O4^ii^	0.86	2.38	3.0908 (17)	140

Symmetry codes: (i) *x*, −*y*+3/2, *z*+1/2; (ii) −*x*+2, *y*+1/2, −*z*+3/2.
