# Supplementary figures and images for: Crystal structure of ethyl 2-amino-4-(4-meth­oxy­phen­yl)-4H-1-benzothieno[3,2-b]pyran-3-carboxyl­ate
Source: Acta Crystallogr E Crystallogr Commun. 2015 Apr 30;71(Pt 5):o366–7. doi: 10.1107/S2056989015008154 (PMC4420120; doi:10.1107/S2056989015008154)

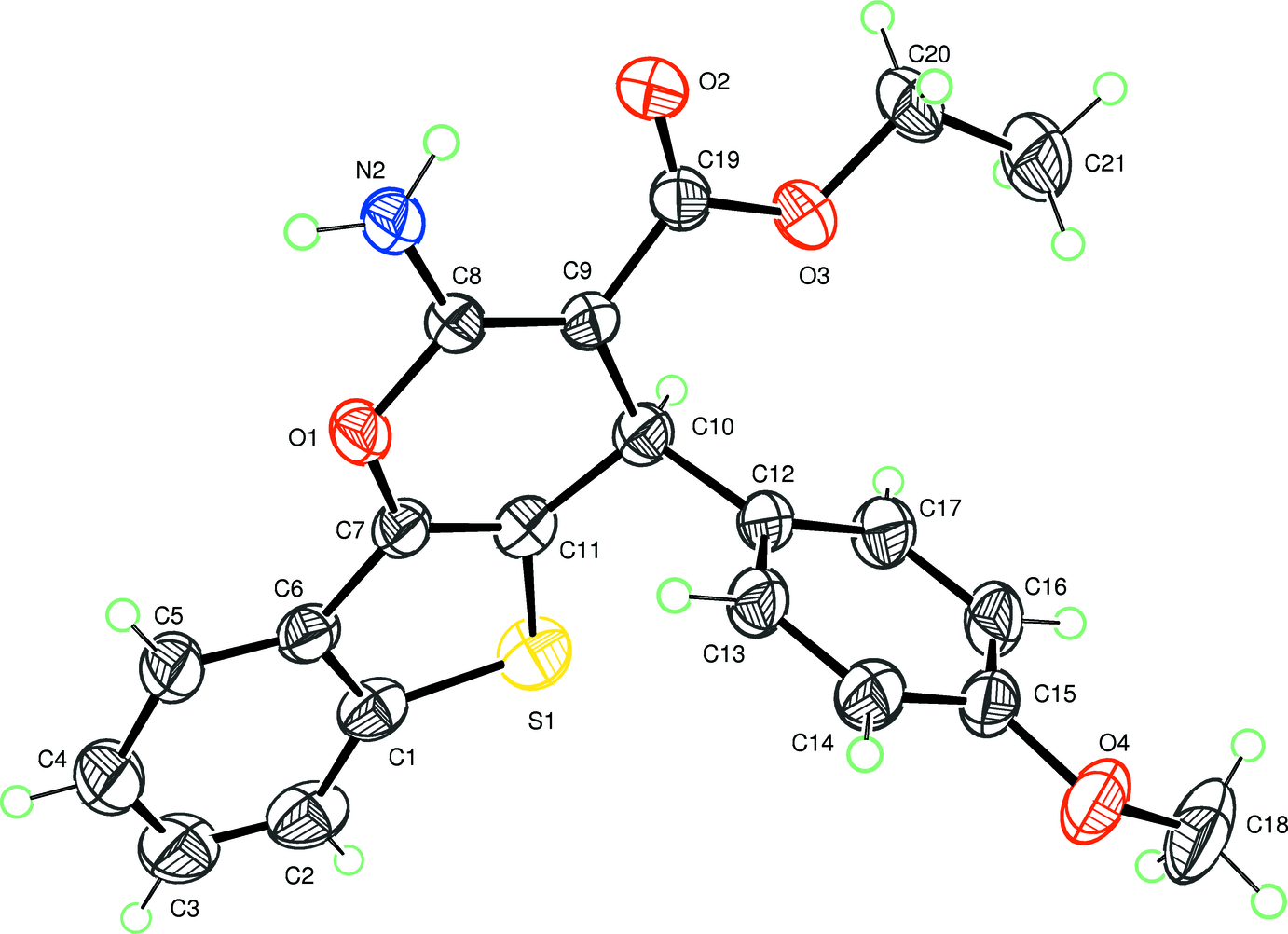

Supplement: Supplementary file 4 [file e-71-0o366-fig1.tif]
